# Artificial Intelligence and Its Revolutionary Role in Physical and Mental Rehabilitation: A Review of Recent Advancements

**DOI:** 10.1155/bmri/9554590

**Published:** 2024-12-17

**Authors:** Amir Rahmani Rasa

**Affiliations:** Department of Occupational Therapy, School of Rehabilitation Sciences, Hamadan University of Medical Sciences, Hamadan, Iran

**Keywords:** artificial intelligence, machine learning, natural language processing, occupational therapy, robotics, virtual reality

## Abstract

The integration of artificial intelligence (AI) technologies into physical and mental rehabilitation has the potential to significantly transform these fields. AI innovations, including machine learning algorithms, natural language processing, and computer vision, offer occupational therapists advanced tools to improve care quality. These technologies facilitate more precise assessments, the development of tailored intervention plans, more efficient treatment delivery, and enhanced outcome evaluation. This review explores the integration of AI across various aspects of rehabilitation, providing a thorough examination of recent advancements and current applications. It highlights how AI applications, such as natural language processing, computer vision, virtual reality, machine learning, and robotics, are shaping the future of physical and mental recovery in occupational therapy.

## 1. Background

Rehabilitation, occupational therapy, and physical therapy are considered interrelated disciplines, sharing a common goal of improving an individual's physical, mental, and emotional abilities following an injury, illness, or disability [1]. According to the World Health Organization, approximately 15% of the world's population lives with some form of disability. This includes physical, cognitive, and emotional disabilities that can significantly impact an individual's daily life and ability to function independently [2]. Regarding the United States, the Centers for Disease Control and Prevention (CDC) have reported more than 61 million adults having some form of disability, with mobility limitations being the most common type. Additionally, the CDC notes that approximately 53 million adults have a mental illness, which can also impact their ability to function in daily life [3].

The staggering numbers of adults with disabilities and mental illness in the United States highlight the critical role of rehabilitation, occupational therapy, and physical therapy in enabling them to regain their physical and cognitive abilities and enhance their quality of life. These interventions involve a complex and integrated approach that aims to maximize the individual's functional capacity, promote independence, and facilitate community integration. Through these therapies, individuals can overcome physical and mental barriers, restore their confidence and self-esteem, and achieve their normal life [1, 4]. Therefore, it is essential to recognize the significance of these disciplines in addressing the healthcare needs of the growing population of patients with disabilities and mental illness. In the following section, we introduce each of these disciplines, highlighting their unique approaches and applications.

Rehabilitation is considered a broad term that encompasses multiple disciplines, including occupational therapy and physical therapy. This field is a systematic and integrated approach that endeavors to restore or develop an individual's lost functions or alternative abilities to adapt to the limitations caused by an injury, illness, or disability [5]. The rehabilitation process begins with a comprehensive assessment of the patient's condition, which helps to identify their strengths and weaknesses, as well as any potential barriers to their recovery. So, based on this assessment, the rehabilitation team (which typically includes physiotherapists, occupational therapists, speech therapists, and rehabilitation physicians) develops a personalized treatment plan that may include a combination of medical management, physical and occupational therapy, speech and language therapy, and cognitive rehabilitation [6, 7].

Occupational therapy, more specifically, aims to help individuals participate in their common daily activities (such as work, self-care, and relief times). In this filed, the therapist works with the individual to improve their ability to perform daily tasks by identifying and considering barriers that may prevent their participation [8]. The therapist may provide training in adaptive techniques, utilize specialized equipment or assistive devices, and implement environmental changes to promote participation in common daily activities. Therefore, the final goal of occupational therapy is to improve an individual's overall quality of life by facilitating their involvement in routine activities [9].

Physical therapy, as another type of rehabilitation, is here to restore, maintain, or improve the physical function and mobility of individuals. Physical therapists work with individuals to develop personalized treatment plans that may include exercises, manual therapy, and other techniques to improve strength, range of motion, balance, coordination, and endurance. Physical therapy may also involve the use of specialized equipment such as braces, crutches, or wheelchairs to improve mobility [10, 11].

Although occupational therapy and physical therapy share common goals, they utilize distinct approaches and techniques. For instance, occupational therapy focuses on enabling individuals to engage in daily activities, whereas physical therapy focuses on recovering, preserving, or enhancing physical function and mobility [12]. Therefore, by understanding the distinct approaches and techniques utilized by each field, healthcare professionals can develop comprehensive and personalized rehabilitation plans to meet the diverse needs of their patients. The advancement of biomedical science, encompassing fields such as digital medicine, artificial intelligence (AI), and its subset, machine learning (ML), sets the stage for a transformative shift in healthcare. This transformation is driven by emerging technologies and necessitates a new type of workforce and updated standards of practice [13]. In physical and mental rehabilitation, these innovations hold significant promise. Moreover, digital health technologies encompass a wide array of tools, including mobile health, health information technology (HIT), virtual reality (VR), wearable devices, telehealth, telemedicine, and personalized medicine. Recently, cutting-edge advancements such as AI, the metaverse, and data sciences have begun to shape the landscape of smart health. These technologies offer easy access to exercises, seamless data integration, real-time monitoring, and remote consultations, enhancing patient adherence and care coordination [14]. Cutting-edge advancements such as AI, the metaverse, and data sciences further personalize and optimize treatment plans, create immersive therapy environments, and improve clinical decision-making through data analysis. This convergence is making rehabilitation more personalized, accessible, and effective [15]. Therefore, this review explores how integrating AI technologies—such as ML, natural language processing (NLP), and computer vision—can revolutionize occupational therapy by enhancing assessment accuracy, personalizing intervention plans, improving treatment efficiency, and evaluating outcomes, providing a comprehensive understanding of AI applications in rehabilitation and future directions for the field.

## 2. AI and Its Importance to Healthcare

AI is a rapidly developing technology that is becoming increasingly important in healthcare. With the ability to analyze large amounts of data, AI has demonstrated the potential to revolutionize healthcare approaches including diagnosis and treatment [16]. This includes various types of medical records, clinical trials, and scientific literature. By analyzing this data, AI algorithms can identify different patterns and trends that may be difficult to analyze or detect for humans (healthcare professionals). This can lead to earlier and more accurate diagnoses, more effective treatments, and more efficient healthcare delivery [17–19].

AI can also help healthcare professionals personalize treatment plans for individual patients. By analyzing patient data such as medical imaging scans (such as x-rays, MRIs, and CT scans) [20–23], laboratory test results (such as blood tests and genetic tests) [24, 25], electronic health records [26, 27], and wearable device data (such as heart rate and activity level) [28], AI algorithms can develop personalized treatment plans that take into account individual differences in disease progression, treatment response, and side effects [29]. Therefore, this personalized approach can lead to better patient outcomes and reduced healthcare costs.

The integration of AI into occupational therapy and physical therapy has the potential to revolutionize the way these therapies are delivered. By providing personalized, data-driven treatment plans, patients can receive more effective and efficient care. Furthermore, the use of AI-powered technologies can enhance patient engagement and motivation, leading to improved outcomes and quality of life [30, 31]. Hence, the aim of the present review is to explore the novel and futuristic applications of AI in rehabilitation, and more specifically occupational therapy.

### 2.1. AI in Occupational Therapy

**Figure 1:**
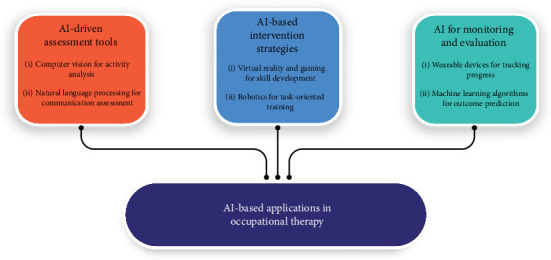
An overview of AI-based application for occupational therapy.

AI can assist occupational therapists in providing more accurate and personalized assessments, interventions, and evaluations to enhance patient outcomes. This includes the use of AI-driven assessment tools, AI-based intervention strategies, and AI for monitoring and evaluation [32]. In the following sections, we will discuss each part with a focus on the role of AI in addressing the unique needs of each patient. Furthermore, Figure 1 provides a summary of AI-based strategies for occupational therapists.

#### 2.1.1. AI-Driven Assessment Tools for Occupational Therapy

AI-driven assessment tools are rapidly emerging technologies with the potential to revolutionize the field of occupational therapy. These tools use sophisticated algorithms and computer vision techniques to provide objective measurements and analysis of movement patterns, daily activities, and functional performance of patients [33]. Moreover, these tools can provide valuable insights into the underlying biomechanics and functional deficits of patients, allowing occupational therapists to develop personalized and effective treatment plans [34]. Among these tools, computer vision and NLP have gained significant attention in recent years.

##### 2.1.1.1. Computer Vision for Activity Analysis

Recent advancements in computer vision have significantly evolved, leveraging AI to improve accuracy and efficiency. The primary aim of computer vision systems is to automatically identify human activities from video data [35]. These systems typically involve several key stages: image capture, segmentation, tracking, identification, and classification. The input is obtained from visual sources like cameras, and human detection is the initial step, involving image preprocessing, segmentation, and feature extraction to distinguish human movements from other objects [36]. Tracking follows various methods such as region-based, contour-based, feature-based, model-based, hybrid, and optical flow–based tracking. Finally, activity recognition is conducted, often utilizing ML algorithms to classify and interpret human actions [37]. The development of computer vision systems is driven by applications in areas such as surveillance, human–computer interaction, and healthcare. Despite numerous advancements, challenges remain, including variations in illumination, camera angles, and accurate object detection. Continuous research focuses on addressing these challenges and enhancing the robustness of computer vision systems [38, 39].  Table 1 provides a summary of recent advancements in vision-based human activity recognition (HAR), highlighting the key techniques, datasets used, strengths, and weaknesses of various methods and systems. These include two-stream convolutional networks, 3D convolutional neural networks (3D CNNs), long short-term memory (LSTM) networks, temporal segment networks (TSNs), attention mechanisms in CNNs, graph convolutional networks (GCNs), transformers, pose-based convolutional networks, self-supervised learning, and hybrid approaches (convolutional neural network+recurrent neural networks (CNN+RNN)). The table highlights the diversity of approaches and the ongoing efforts to improve the accuracy, efficiency, and applicability of computer vision systems in real-world scenarios.

In occupational therapy, hopefully, computer vision can be used to analyze patients' movement patterns and daily activities to provide insights into their functional performance. In this case, one application of computer vision algorithms can be the capability to track the movement of patients during tasks such as reaching, grasping, and lifting to identify any deviations from normal movement patterns [52]. This technology, in combination with motion capture as well as AI, can provide detailed information on joint angles, range of motion, and movement patterns, allowing therapists to identify areas of weakness or dysfunction [53].

There are several commercial examples of these technologies in occupational therapy. In this regard, the Vicon motion capture system, as a commercial product for motion capture and analysis, has been used to assess movement patterns and functional performance in patients with conditions such as stroke, cerebral palsy [54], and spinal cord injuries [55]. For instance, in a study by Yavuzer et al., they investigated the potential use of the Vicon motion capture system for assessing movement patterns and functional performance in patients with stroke. Regarding this, the results from their study have demonstrated that the Vicon system was effective in measuring joint angles and movement patterns during various tasks such as walking and stair climbing. The system provided accurate and reliable data on patients' functional performance, including gait speed, step length, and stride duration. They have concluded that the Vicon system has potential as a clinical tool for assessing and monitoring the progress of stroke patients during rehabilitation by providing objective data on the patient's functional performance, allowing therapists to track their progress and adjust their treatment plans accordingly. Therefore, this study showed the potential benefits of using motion capture technology in stroke rehabilitation, which supports the use of commercial products such as the Vicon system for this purpose [56].

Microsoft Kinect is another computer vision product that is used to develop therapeutic games and exercises for patients with various conditions in occupational therapy such as stroke [57], traumatic brain injury [58], and Parkinson's disease [59]. In these studies, researchers have demonstrated the potential of Microsoft Kinect as a tool in occupational therapy to improve functional performance and promote recovery in patients with various conditions. Although products like the Vicon motion capture system and Microsoft Kinect are not considered AI systems, the data that is captured by this system can be very robust and detailed, providing information on the movement of individual joints, limbs, and even entire bodies. This data can then be used as input for ML algorithms, which can analyze the movement patterns and make predictions or decisions based on the data [57, 59].

Computer vision based on HAR in occupational and physical therapy employs a variety of methods and systems to monitor and analyze patient movements, improving therapy outcomes [60]. These include sensor-based systems like wearable sensors, vision-based systems using RGB and depth cameras, and hybrid systems combining both [61]. ML techniques, such as supervised learning and deep learning models like CNNs and LSTMs, provide robust automatic feature extraction and temporal pattern recognition [62, 63]. Computer vision techniques, including optical flow and pose estimation tools like OpenPose, offer detailed motion and posture information [64]. Hybrid approaches that fuse sensor and vision data enhance accuracy but increase complexity. Effective data processing, feature extraction, and data augmentation are essential for accurate HAR. Evaluation methods like cross-validation ensure reliable performance, while telerehabilitation platforms increase therapy accessibility and enable continuous monitoring, despite privacy and connectivity challenges [65]. Here is Table 2. We have an overview of the recent technologies used in occupational and other related fields.

##### 2.1.1.2. NLP for Communication Assessment

In addition to computer vision, NLP is another AI technology that possesses the capacity to fundamentally transform occupational therapy. Overall, NLP is a technique that enables computers to understand and interpret human language [72]. In occupational therapy, NLP can be used to assess patients' communication abilities and provide personalized interventions to improve their functional communication skills. In this regard, in several studies, the potential of NLP-based tools has been evaluated. For instance, in a study conducted by Agaronnik et al., they showed that the LanguageLab system was effective in improving communication skills and reducing communication barriers for patients with aphasia. The system uses NLP algorithms to analyze patients' spoken language and provide personalized feedback on areas for improvement. The results of their study showed significant improvements in communication abilities, including increased fluency and accuracy of speech, improved comprehension of spoken language, and increased confidence in communication. Therefore, the LanguageLab system was found to be effective in providing personalized feedback and encouraging patients to practice and improve their communication skills [73].

Moreover, Yeung et al. conducted a study where clinicians rated speech characteristics of audio recordings from 30 participants with Alzheimer's dementia (AD), mild cognitive impairment (MCI), and controls. The speech recordings were transcribed, and linguistic and acoustic variables were extracted using NLP and automated speech analysis (ASA). The correlations between clinician-rated speech characteristics and the variables were compared using Spearman's correlation, and exploration factor analysis was applied to find common factors between variables for each speech characteristic. The results showed that the variables extracted through NLP and ASA were significantly correlated with clinician ratings of speech and language characteristics, indicating that this approach could be an objective and innovative way to measure speech and language changes in neurodegenerative disorders [74].

Therefore, NLP can also help occupational therapists identify patterns and trends in patient data, allowing them to make more informed clinical decisions. While there are still limitations and challenges to overcome, such as the need for large and diverse datasets and the potential for bias in ML algorithms, NLP is constantly evolving and improving. With further research and development of NLP pipelines, it has the potential to revolutionize communication assessment in speech and occupational therapy approaches and improve the quality of care for patients [75]. In the table (Table 3), we have comprehensively listed various methods and systems for NLP used in communication assessment within occupational and physical therapy, detailing their descriptions, advantages, and disadvantages.

#### 2.1.2. AI-Based Intervention Strategies

As the field of occupational therapy continues to evolve, AI-based intervention strategies are emerging as a promising approach to enhance patient outcomes. These strategies utilize the power of AI to analyze patient data and provide personalized interventions that address individual needs. Two examples of AI-based intervention strategies in occupational therapy include VR and gaming for skill development and robotics for task-oriented training. By incorporating these innovative approaches, occupational therapists can provide more effective and engaging interventions that promote functional improvement and independence for their patients [90].

##### 2.1.2.1. VR and Gaming for Skill Development

VR and gaming are innovative tools that have been used in occupational therapy for skill development. VR technology creates a simulated environment that provides a sense of presence, allowing patients to engage in virtual activities that can improve their physical, cognitive, and emotional function. Similarly, gaming technology provides an interactive and engaging platform that motivates patients to participate in therapy and can enhance their overall experience [91, 92]. Using AI, therapists can also develop simulations that mimic real-world environments and activities, providing patients with a safe and controlled environment in which to practice and improve their skills.

In occupational therapy, VR therapy can be used to help individuals with physical disabilities or injuries regain strength and mobility. By providing simulated environments that mimic real-world tasks such as lifting objects, reaching for items, or navigating through obstacles, patients can work on their physical skills in a safe and controlled environment [93]. Similarly, VR therapy can be used to help individuals with cognitive impairments, such as memory loss, practice tasks related to memory and attention [94].

Occupational therapy practitioners can leverage the potential of VR in treatment sessions by enabling clients to engage in a high dose of therapy or repetition early in the recovery process. According to Kiper et al.'s study, in a typical occupational therapy session for upper extremity rehabilitation following a stroke, clients would only perform 23–32 movements of the affected upper limb. In contrast, VR interventions resulted in up to 200–300 functional upper limb movements completed in a 1-h session. VR has demonstrated the ability to promote greater repetition of movements, leading to faster motor skill acquisition and recovery, while also distracting clients from the realization that they are repeating the same motions [95]. In another study by Botella et al., they studied the use of virtual reality exposure–based therapy (VR-EBT) as a potential treatment for posttraumatic stress disorder (PTSD). In this regard, they conducted a review of existing literature and found that VR-EBT is effective in treating PTSD, but there is a need for more controlled studies and standardization of treatment protocols. Additionally, they suggest that more research is needed to determine the acceptability of VR-EBT for patients with PTSD. Despite these limitations, the study highlights the potential of VR-EBT for PTSD treatment and offers suggestions for future research in this area [96].

Additionally, VR and gaming can be adapted to meet the unique needs of individual patients. VR offers the advantage of easy gamification, where a task or activity can be transformed into an experience that incorporates the positive aspects of games. This process is called gamification, which aims to promote the enjoyment, skills, and practices associated with gaming [97]. VR can create semi-immersive virtual worlds, like the Wii gaming system, which can be tailored to promote specific functional capacities while engaging in desired occupations, such as racing through the Grand Canyon on a kayak, playing catch with a favorite quarterback, or building an exotic garden in a fantasy world. These engaging VR sessions can improve motivation to participate in therapy, potentially reducing burnout during the therapeutic process [98].

In another study conducted by Roberts et al., they aimed to assess the acceptability and effects of a pediatric constraint–induced movement therapy camp augmented by the Hocoma Armeo Spring Pediatric exoskeleton and VR games for children with hemiplegic cerebral palsy (hCP). The results of the study demonstrated that the intervention was both feasible and accepted by the participants. Moreover, the participants showed significant improvement in bimanual performance and occupational performance, and the treatment effects persisted for up to 6 months after the intervention. Moreover, they highlight the potential of incorporating VR games and exoskeletons into occupational therapy interventions for children with hCP. The use of these technologies may provide a more engaging and enjoyable experience for the children, which could increase their motivation to participate in therapy and ultimately lead to better treatment outcomes. Additionally, the study has shown the importance of measuring both clinical and functional outcomes in assessing the effectiveness of interventions. While the improvement in unilateral performance was not clinically significant, the improvement in bimanual performance and occupational performance suggests that the intervention had a meaningful impact on the children's daily activities and participation [99].

Taken together, the use of VR technology in rehabilitation is a promising new approach that has been shown to improve outcomes in comparison to traditional methods. VR is not only a facilitator of practice but also an integral part of our daily lives that enhances our interactions with the environment. It can be utilized for a wide range of diagnoses and across all ages to simulate, immerse, expose, and encourage the desired therapeutic results at a relatively cost-effective price. Occupational therapy practitioners should evaluate the feasibility of integrating this technology into their practices and assess the technical development of these tools to ensure that they produce the desired outcomes for the clients while promoting compliance and reducing the monotony associated with conventional rehabilitation interventions [100]. In summary, Table 4 provides a comprehensive overview of methods and systems used in VR and gaming for skill development in related fields of occupational, rehabilitation, and physical therapy.

##### 2.1.2.2. Robotics for Task-Oriented Training

Occupational therapists have been incorporating robotics into their treatment plans for a long time, especially when working with younger clients who benefit from play-based interventions. However, in recent years, advancements in technology have allowed robotics to become more sophisticated and versatile in their applications. In particular, AI has enabled robots to interact with people across the lifespan, providing social engagement and tailored experiences [32].

Robots equipped with image recognition and ML capabilities can now recognize and respond to specific individuals' needs and preferences [112]. For example, Paro, a plush seal robot, has been designed to serve as a social companion for older adults. With dual 32-bit processors, microphones, tactile sensors, touch-sensitive whiskers, and motors and actuators that move their limbs and body, Paro can respond to touch, sound, and light. The robot has sensors that detect touch, allowing it to cuddle with people and remember faces. It actively seeks eye contact, responds to sounds, and can learn names, including its own. To enhance the user experience, Paro can produce sounds like a real baby seal, making it feel like a living creature. Its advanced AI system allows it to learn and adapt to the user's behavior and personality, providing a personalized and interactive experience [113].

Moreover, Liang et al. conducted a study to investigate the effects of Paro on people with dementia in both a day care center and a home setting. The results indicated that Paro significantly improved facial expressions and communication with staff at the day care center. The robot's behavioral and physiological effects were also assessed, and it was found that Paro reduced the number of negative behaviors in people with dementia, such as agitation and wandering. Paro's ability to recognize and customize its behavior to the end user makes it an ideal assistive technology for people of all ages. With its advanced features, Paro can provide emotional support, improve communication, and reduce negative behaviors in people with various conditions, including dementia. The integration of robotics and AI in occupational therapy can offer new and innovative ways to enhance the quality of life for clients [113].

Table 5 provides a comprehensive overview of robotics methods and systems utilized for task-oriented training in occupational, rehabilitation, and physical therapy. Specialized rehabilitation robots focus on gait and limb recovery, while socially assistive robots enhance patient engagement. Integration with VR and telerehabilitation systems broadens accessibility. Adaptive robots adjust assistance based on real-time feedback, and cognitive training robots combine physical and mental exercises. Data analytics tools enable detailed performance monitoring, and gamification approaches use game elements to boost motivation. Each method offers distinct advantages in patient engagement and therapeutic effectiveness but also presents challenges related to cost, complexity, and technical requirements.

Therefore, the integration of AI and robotics in occupational therapy practices can provide numerous benefits for clients. Robots can assist in therapy sessions, providing customized feedback and encouragement to help clients reach their goals [126]. They can also provide a source of social interaction, reducing loneliness and isolation, especially for older adults [127].

#### 2.1.3. AI for Monitoring and Evaluation

The field of occupational therapy is continuously evolving, and the integration of technology is playing a significant role in improving patient outcomes. One of the latest technological advancements in this field is the use of AI to track progress and evaluate the effectiveness of interventions [128]. In recent years, the integration of wearable devices and ML-based algorithms has become increasingly popular to enhance patient assessment and treatment outcomes. Wearable devices, such as fitness trackers and smartwatches, can provide real-time data on a client's movement patterns, vital signs, and daily activities [129]. ML algorithms can then analyze this data and provide predictions on future outcomes, allowing occupational therapists to make more informed decisions about their client's treatment plans [128]. This combination has the potential to revolutionize the way occupational therapy is practiced, leading to more personalized and effective interventions.

##### 2.1.3.1. Wearable Devices for Tracking Progress

Wearable technology has emerged as a game-changer in the field of occupational therapy, offering a new way to track and monitor clients' health and progress. With the use of smartwatches, body-mounted sensors, and fitness trackers, occupational therapists can collect valuable data on various biometric measures, including the number of steps taken, blood pressure, breathing patterns, heart rate, and sleep quality [130]. By pairing these devices with an app that stores biometric data, occupational therapists can easily monitor and evaluate clients' progress, enabling them to develop more effective treatment plans that are tailored to individual needs. This use of wearable technology has reduced the need for trial-and-error treatments and has ushered in a more personalized and data-driven approach to occupational therapy [131].

Generally, wearable devices can be used in various aspects of occupational therapy, including (1) assessment and data collection: wearable devices can provide objective data on physical activity, movement quality, and vital signs, helping therapists better understand their patients' needs and track their progress over time [132]; (2) biofeedback and self-monitoring: real-time feedback from wearable devices can help patients become more aware of their body and movement patterns, promoting self-regulation and improving motor control [133]; (3) motivation and engagement: gamification and interactive features in wearable devices can encourage patients to participate in therapy and adhere to their treatment plan [134]; (4) telehealth and remote monitoring: wearable devices can enable therapists to monitor patients' progress and provide remote support, reducing the need for in-person appointments [135];and (5) personalized interventions: data collected from wearable devices can help therapists tailor their treatment approach, targeting specific areas of difficulty and customizing the intervention according to the patient's needs and preferences [136].

In Table 6, we provide a comprehensive overview of wearable devices utilized for tracking progress in occupational, rehabilitation, and physical therapy. This overview is aimed at aiding in understanding the diverse tools available for enhancing therapy effectiveness and patient progress monitoring.

##### 2.1.3.2. ML Algorithms for Outcome Prediction

Healthcare research has effectively demonstrated the use of wearable sensors in various applications, including continuous monitoring of activities of daily living, gait, and mobility [147, 148]. However, the growing usage of wearable sensors presents a significant challenge for data analysis, as they record large amounts of time-series data. Nevertheless, the rapid development of data analytic methods has enabled vast amounts of data to be processed, uncovering hidden information [149, 150]. In recent years, there has been an increased interest in using ML algorithms to improve the outcome prediction, monitoring, and evaluation of occupational therapy interventions. On the other hand, ML algorithms can be used to analyze data from various sources, including patient records, assessments, and sensor data, to predict the outcomes of interventions. In this regard, Yang et al. have created an innovative hand function recovery system using a smart wearable armband that utilizes surface electromyography to detect biopotential signals and ML algorithms to recognize various hand movement patterns. The system is coupled with a dexterous robot hand that can mimic the user's hand gestures. By analyzing the sensor data with ML algorithms, the system can provide targeted interventions, which is a promising technology for automating and improving the quality of intelligent decision-making in healthcare service delivery [151].

Recent evidence has shown that ML can also be used for outcome assessments in addition to interventions. For example, a client's performance during a rehabilitation exercise can be classified based on whether they are executing the given exercise correctly. This highlights the potential of ML as a tool for accurately and objectively evaluating the effectiveness of rehabilitation interventions [152]. To assess spasticity, wearable devices that measure biomechanical data from multiple sensors, such as force and angle sensors, have been integrated with artificial neural networks. By learning patterns from this data, neural networks can provide accurate spasticity assessments [153, 154]. Zhang et al. employed supervised learning algorithms based on regression analysis to predict the Modified Ashworth Scale (MAS) scores using data collected from electromyography signals and wearable devices equipped with a triaxial accelerometer, gyroscope, and magnetometer [155]. In another study, Kim et al. aim to develop a method to determine the severity of elbow spasticity by analyzing acceleration and rotation data from a wearable device and using ML algorithms to classify the degree of spastic movement. The proposed approach is comparable to assigning a MAS score and can provide patients with the ability to monitor spasticity outside of healthcare institutions. The study collected inertial data from participants using a wearable device and evaluated various ML algorithms. A random forest algorithm performed well, achieving up to 95.4% accuracy. This research highlights the potential of wearable technology and ML in generating clinically meaningful indices for rehabilitation patients [149].

Therefore, ML algorithms have the potential to improve outcome prediction in occupational therapy by analyzing large amounts of patient data and identifying patterns that may not be immediately apparent to human clinicians [81]. However, for these algorithms to be effective, they must be trained on high-quality data that accurately represents the patient population of interest [156]. Additionally, it is important for clinicians to work closely with data scientists and ML experts to ensure that the algorithms are being used in an ethical and responsible manner. With careful implementation and ongoing evaluation, ML algorithms have the potential to significantly improve the quality of care provided to occupational therapy patients.

## 3. Current Interdisciplinary Trends and Future Directions for AI Applications in Rehabilitation

The integration of AI in occupational therapy has shown promising results in assessment, intervention, and evaluation, and there are several areas where further research and development could lead to significant advancements, which include the following:

• Expanding AI-driven assessment tools: Future research should focus on developing new AI-driven assessment tools that can address a broader range of patient needs, such as sensory integration, cognitive skills, and fine motor skills. These tools could also be designed to accommodate various age groups, cultural backgrounds, and clinical settings [157].
• Improving AI-based intervention strategies: The use of VR, gaming, and robotics in occupational therapy interventions has shown potential, but further exploration is needed to determine the most effective strategies for various patient populations. This could include incorporating more realistic simulations, designing adaptive games that adjust to individual needs, or developing smarter robotic assistants capable of learning from patient interactions [158].
• Enhancing AI for monitoring and evaluation: As wearable devices and ML algorithms continue to improve, research should focus on refining these tools to accurately capture patient progress and predict outcomes. This can be achieved by incorporating more comprehensive datasets, improving the accuracy of predictive models, and developing interfaces that allow occupational therapists to easily interpret and utilize the data [159].
• Addressing ethical and privacy concerns: As AI becomes more integrated into healthcare, it is essential to consider the ethical implications and privacy concerns that arise. Future research should focus on developing guidelines and best practices for maintaining patient confidentiality, consent, and autonomy while using AI-driven tools in occupational therapy [160].
• Interdisciplinary collaboration: To further advance the role of AI in occupational therapy, collaboration between computer scientists, engineers, occupational therapists, and other healthcare professionals is essential. Such cooperation will help to ensure that AI-driven tools and strategies are developed and implemented with a comprehensive understanding of both the technical and clinical aspects involved [161].

The utility of AI as a benchmark resource can be further enhanced by addressing emerging interdisciplinary trends and their future applications in rehabilitation. Strengthening its role in this way would offer more practical guidance for the community, especially considering the rapidly evolving landscape of AI in healthcare. To begin with, the focus could be on current interdisciplinary trends in healthcare AI that span not just rehabilitation but broader fields like wearable technologies, robotics, and neuroengineering [162, 163]. Moreover, emerging technologies such as brain–computer interfaces, advanced biosensors, and real-time monitoring systems integrated with AI are pivotal for creating personalized rehabilitation plans. Expanding the discussion to cover these interdisciplinary trends would help the community understand how AI-based rehabilitation models could be integrated into the growing ecosystem of digital health, allowing seamless data sharing and analysis across platforms. This approach would also align AI development with personalized and precision medicine, which are key directions for the future [163, 164]. Additionally, addressing future directions for AI in rehabilitation should include exploring solutions for existing interdisciplinary challenges, such as data interoperability and the ethical implications of AI deployment. For instance, standardizing data collection methods and formats across disciplines (e.g., wearable sensors, imaging, and electronic health records) is crucial for developing robust AI models that can adapt to various clinical scenarios. Furthermore, the field could benefit from examining the regulatory landscape and the ethical dimensions of AI in rehabilitation, particularly regarding data privacy, security, and the transparency of AI-driven decisions [61, 165].

Finally, focusing on potential collaborative opportunities between AI developers, clinicians, and policymakers could pave the way for more successful and ethically grounded AI integration into rehabilitation practices. This would ensure that the models developed are clinically actionable, reliable, and capable of addressing real-world problems in a scalable manner, thereby solidifying the paper's role as a forward-looking benchmark for the community. By expanding on these interdisciplinary and future-oriented dimensions, we could further elevate the practical relevance of their work in advancing AI applications in rehabilitation medicine [163, 166].

## 4. Conclusion

AI has the potential to revolutionize occupational therapy by enhancing the accuracy and personalization of assessments, interventions, and evaluations. With continued research and development in AI-driven assessment tools, AI-based intervention strategies, and AI for monitoring and evaluation, occupational therapists can further improve patient outcomes and address the unique needs of everyone. By collaborating with experts across various disciplines and addressing ethical and privacy concerns, we can ensure that the integration of AI in occupational therapy is both effective and responsible.

## Figures and Tables

**Table 1:** Recent methods and systems for computer vision using artificial intelligence.

**Category**	**Method/system**	**Description**	**Advantages**	**Disadvantages**	**Ref.**
Two-stream convolutional networks	Spatial and temporal stream fusion	UCF101, HMDB51	Good performance in action recognition	High computational cost, requires pretraining	[40, 41]
3D convolutional neural networks (3D CNNs)	3D convolutions over spatial and temporal dimensions	Sports-1M, Kinetics-400	Effective for capturing spatiotemporal features	High computational requirements	[42, 43]
Long short-term memory (LSTM) networks	Sequence learning with LSTM	NTU RGB+D, MSR Action3D	Effective for temporal dependencies	Can be slow to train	[44]
Temporal segment networks (TSNs)	Sparse temporal sampling	UCF101, HMDB51	Efficient and accurate	May miss fine-grained details in action	[45]
Attention mechanisms in CNNs	Focused feature extraction	Kinetics-400, something-something	Improves recognition by focusing on important features	Computationally intensive, complex implementation	[46]
Graph convolutional networks (GCNs)	Graph-based representation	NTU RGB+D, Kinetics-400	Effective for modeling relationships between body parts	Requires complex preprocessing	[47]
Transformers for HAR	Self-attention mechanisms	Kinetics-600, HACS	Captures long-range dependencies, scalable	High memory consumption	[48]
Pose-based convolutional networks	Keypoint detection and analysis	COCO, MPII	Effective for actions involving body poses	Limited by the accuracy of pose estimation	[49]
Self-supervised learning for HAR	Unsupervised pretraining	Kinetics-400, UCF101	Reduces the need for labeled data	Often requires fine-tuning with labeled data	[50]
Hybrid approaches (CNN+RNN)	Combined CNN and RNN architectures	NTU RGB+D, HMDB51	Leverages strengths of both CNNs and RNNs	Increased complexity and training time	[51]

Abbreviations: CNN, convolutional neural network; COCO, Common Objects in Context; GCN, graph convolutional network; HAR, human activity recognition; LSTM, long short-term memory; MPII, Max Planck Institute for Informatics; TSNs, temporal segment networks.

**Table 2:** Methods and systems for computer vision in occupational and physical therapy.

**Category**	**Method/system**	**Description**	**Advantages**	**Disadvantages**	**Ref.**
Vision-based systems	RGB cameras	Use of regular cameras to capture color images and videos of patients performing activities.	Easy to set up; provides rich visual data.	Privacy concerns; sensitive to lighting and occlusion issues.	[66]
Depth cameras	Kinect	Utilizes infrared light to capture depth information, enabling 3D reconstruction of the scene.	Provides detailed 3D data; robust to lighting conditions.	Limited range; can struggle with reflective surfaces.	[67]
Hybrid systems	RGB-D cameras	Combines RGB and depth sensors to capture both color and depth information simultaneously.	Combines advantages of both RGB and depth cameras; enhanced feature extraction.	Higher cost; complex data processing.	[68]
Computer vision techniques	Optical flow	Measures motion between two consecutive frames to detect movement.	Provides detailed motion information; useful for dynamic activities.	Sensitive to noise and fast movement; computationally expensive.	[69]
Pose estimation	OpenPose	Detects and tracks key points on the human body to understand posture and movement.	Provides detailed body posture information; real-time processing.	Can struggle with occlusions and complex poses; computationally intensive.	[70]
Hybrid approaches	Fusion of sensors and vision	Combines data from wearable sensors and vision-based systems to enhance activity recognition.	Combines the strengths of both approaches; improved accuracy and robustness.	High complexity; requires synchronization of data from different sources.	[71]

**Table 3:** Methods and systems for natural language processing in communication assessment for occupational and physical therapy.

**Category**	**Method/system**	**Description**	**Advantages**	**Disadvantages**	**Ref.**
Speech recognition	Automatic speech recognition (ASR)	Converts spoken language into text using algorithms and machine learning.	Facilitates real-time analysis; improves accessibility for nonverbal patients.	Can struggle with accents, dialects, and background noise; requires substantial training data.	[76]
Text analysis	Sentiment analysis	Analyzes text to determine the sentiment (positive, negative, and neutral) expressed by the speaker.	Provides insights into patient emotions and mental state.	May misinterpret context and sarcasm; relies on the quality of input text.	[77]
Syntax and semantics	Part-of-speech (POS) tagging	Identifies and labels parts of speech in text (nouns, verbs, adjectives, etc.).	Useful for analyzing grammatical structure; aids in language development assessment.	Can be inaccurate with complex sentences; requires high-quality linguistic models.	[78]
Discourse analysis	Topic modeling	Identifies topics discussed in text using algorithms like latent Dirichlet allocation (LDA).	Helps understand conversation themes and patient interests.	Can oversimplify or misclassify topics; sensitive to input text quality.	[79]
Pragmatics	Dialogue systems	Utilizes chatbots and virtual assistants to engage patients in conversation, assessing their communication skills.	Provides consistent, scalable interaction; can be tailored to specific therapy goals.	May lack human empathy and adaptability; effectiveness depends on the quality of the conversational model.	[80]
Machine learning	Supervised learning	Uses labeled datasets to train models for specific communication assessments.	High accuracy with adequate training data; adaptable to various assessment needs.	Requires large, labeled datasets; can be time-consuming to train.	[81]
Deep learning	Recurrent neural networks (RNNs)	Models' sequential data, capturing temporal dependencies in speech and text for nuanced communication analysis.	Effective for analyzing sequential patterns; handles complex language structures.	Computationally intensive; requires significant computational resources.	[82]
Feature extraction	Text embeddings (e.g., Word2Vec, BERT)	Convert text into numerical vectors, preserving semantic relationships for further analysis.	Captures semantic nuances; facilitates advanced text analysis.	Can be resource-intensive; requires large corpora for effective training.	[83]
Evaluation	Cross-validation	Splits data into training and testing sets to validate NLP model performance.	Ensures reliable performance estimates; helps prevent overfitting.	Computationally intensive; maybe time-consuming.	[84]
Application-specific systems	Teletherapy platforms	Remote therapy systems that incorporate NLP tools to assess and enhance patient communication skills during online sessions.	Increase accessibility; provide continuous monitoring and feedback.	Requires stable internet connection; potential privacy issues.	[85]
Data augmentation	Synthetic data generation	Creates synthetic speech and text data to augment training datasets, improving model robustness and performance.	Enhances model robustness; helps overcome data scarcity.	May introduce unrealistic data; requires careful design to ensure quality.	[86]
Multimodal systems	Speech and gesture integration	Combines speech recognition with gesture analysis to provide a comprehensive assessment of communication abilities.	More holistic understanding of communication skills; addresses limitations of single-modality systems.	High complexity; requires integration of diverse data sources.	[87]
Real-time processing	Stream processing	Analyzes speech and text data in real-time to provide immediate feedback and assessment during therapy sessions.	Facilitates timely interventions; enhances interactive therapy experiences.	Requires high computational power; may have latency issues.	[88]
Personalized therapy	Adaptive learning systems	Utilizes patient data to tailor communication assessments and interventions to individual needs, ensuring personalized therapy plans.	Enhances therapy effectiveness; caters to individual patient progress.	Requires comprehensive patient data; potential privacy and data security concern	[89]

**Table 4:** Methods and systems for virtual reality and gaming in skill development for occupational and physical therapy.

**Category**	**Method/system**	**Description**	**Advantages**	**Disadvantages**	**Ref.**
Virtual reality systems	Immersive VR	Fully immersive environments using VR headsets to simulate real-world scenarios for therapy and skill development.	High level of engagement; realistic simulations; customizable environments.	Expensive equipment, potential for motion sickness; requires technical expertise.	[101]
Nonimmersive VR	Desktop VR	Uses standard computer screens to display VR environments, allowing interaction through keyboard, mouse, or other input devices.	More accessible and affordable than immersive VR; easier to implement.	Less immersive; may not provide the same level of engagement.	[102]
Augmented reality (AR)	Mobile AR	Uses smartphones or tablets to overlay digital information on the real world, aiding in skill development through interactive exercises.	Portable and accessible; combines real and virtual elements.	Limited device capabilities can be less immersive.	[103]
Serious games	Therapy-focused games	Custom-designed video games aimed at developing specific skills and facilitating rehabilitation exercises.	Engaging and motivating; can be tailored to individual therapy goals.	Development can be costly and time-consuming; effectiveness depends on game design quality.	[104]
Gamification	Point and reward systems	Incorporates game-like elements (e.g., points, badges, and leaderboards) into therapy exercises to increase motivation and participation.	Enhances motivation and engagement; provides immediate feedback.	May not be suitable for all patients; risk of overemphasis on rewards rather than skill development.	[105]
Motion tracking	Kinect-based systems	Use motion-sensing devices to track body movements, enabling interaction with virtual environments and games.	Provides real-time feedback; supports a wide range of movements.	Limited precision; requires adequate space and setup.	[106]
Haptic feedback	Haptic gloves	Utilize gloves or other wearables that provide tactile feedback, enhancing interaction with virtual environments.	Enhancing realism and immersion helps develop fine motor skills.	It can be expensive; it may require significant setup and calibration.	[107]
Cognitive training	VR cognitive games	Games designed to improve cognitive functions such as memory, attention, and problem-solving within a VR environment.	Provides a stimulating and controlled environment for cognitive training.	May not address physical rehabilitation needs; require cognitive engagement.	[108]
Multiuser VR	Collaborative VR environments	Enables multiple users to interact within the same virtual space, promoting social skills and teamwork in therapy.	Encourages social interaction and teamwork; can simulate group activities.	Requires robust network infrastructure; potential for technical issues.	[109]
Remote VR therapy	Tele-VR	Delivers VR-based therapy sessions remotely, allowing patients to engage in rehabilitation exercises from home.	Increases accessibility; allows for continuous therapy outside clinical settings.	Requires reliable internet connection; potential privacy concerns.	[110]
VR simulation	ADL simulation	Simulates activities of daily living (ADL) in a virtual environment to help patients practice and develop necessary skills.	Realistic practice scenarios; customizable to patient needs.	May require extensive programming and scenario development.	[111]
VR-based biofeedback	Physiological monitoring	Integrates biofeedback sensors with VR systems to monitor physiological responses (e.g., heart rate and muscle tension) during therapy.	Provides real-time physiological data can enhance the effectiveness of therapy.	Requires specialized equipment; may be intrusive for some patients.	[31]

**Table 5:** Methods and systems for robotics in task-oriented training for occupational and physical therapy.

**Category**	**Method/system**	**Description**	**Advantages**	**Disadvantages**	**Ref.**
Assistive robotics	Exoskeletons	Wearable robotic devices that assist or enhance limb movement, supporting task-oriented training and rehabilitation exercises.	Provides precise assistance; enhances strength and endurance.	High cost; bulky; may require customization to fit individual patients.	[114]
End-effector robots	Robotic arms	Robots that interact with patients through a single contact point, typically at hand or foot, guiding and assisting in task-specific movements.	High precision in movement; adaptable to various tasks.	Limited to distal limb training can be expensive.	[115]
Mobile robots	Autonomous mobile robots	Robots that navigate through the environment autonomously and assist patients in performing tasks and activities.	Enhances mobility training; can simulate real-world scenarios.	Requires advanced navigation systems; potential safety concerns.	[116]
Haptic robots	Haptic feedback devices	Robots provide force feedback to the user, enabling realistic interaction with virtual environments or physical objects.	Enhances sensory feedback; aids in fine motor skill development.	It can be costly; requires precise calibration and maintenance.	[117]
Rehabilitation robots	Gait training robots	Specialized robots are designed to assist with gait training, helping patients to walk and improve their walking patterns through repetitive, task-oriented exercises.	Provides consistent and repetitive training; can adjust to patient's progress.	High cost; requires specialized setup and space.	[118]
Socially assistive robots	Interactive robots	Robots are designed to interact socially with patients, providing motivation, engagement, and companionship during therapy.	Enhances patient motivation and engagement; can be personalized to patient needs.	Limited to social interaction tasks; effectiveness depends on the robot's design and interaction capabilities.	[119]
Upper limb rehabilitation	Arm rehabilitation robots	Robots that assist with the rehabilitation of upper limb function, focusing on task-oriented exercises to improve arm, wrist, and hand movements.	Provides targeted upper limb therapy; can track and adapt to patient progress.	Expensive; it may require extensive setup and space.	[120]
Lower limb rehabilitation	Leg rehabilitation robots	Robots that aid in the rehabilitation of lower limb function, focusing on exercises that enhance leg strength, coordination, and mobility.	Supports lower limb recovery; provides consistent and controlled exercises.	High cost; large footprint; may require professional supervision.	[121]
Virtual reality integration	VR-enhanced robotics	Combines robotics with virtual reality to create immersive, interactive training environments that enhance task-oriented therapy.	Immersive experience; enhances engagement and motivation.	Requires complex integration; high cost; potential for technical issues.	[122]
Telerehabilitation robots	Remote-controlled robots	Robots are controlled remotely by therapists to deliver rehabilitation exercises and monitor patient progress, allowing therapy to be conducted outside clinical settings.	Increases accessibility; enables remote monitoring and adjustment by therapists.	Requires reliable internet connection; potential privacy and security concerns.	[123]
Cognitive training robots	Robotic cognitive trainers	Robots are designed to provide cognitive training through task-oriented activities, enhancing both physical and cognitive rehabilitation.	Enhance cognitive and physical training simultaneously; interactive and engaging.	Limited to tasks that involve both cognitive and physical elements; can be complex to design and implement.	[124]
Gamification	Game-based robotic systems	Incorporates gaming elements into robotic therapy systems to enhance patient engagement and motivation during task-oriented training.	Increases patient motivation; makes therapy sessions enjoyable.	Development and integration can be costly and time-consuming; effectiveness depends on game design.	[125]

**Table 6:** Methods and systems for wearable devices in tracking progress for occupational and physical therapy.

**Category**	**Method/system**	**Description**	**Advantages**	**Disadvantages**	**Ref.**
Activity trackers	Fitness bands	Wearable bands that monitor physical activity, including steps taken, distance traveled, and calories burned.	Easy to use; provides basic activity metrics; affordable.	Limited to activity tracking; may not capture detailed rehabilitation progress.	[137]
Smartwatches	Multifunction watches	Wearable devices with capabilities to track physical activity, monitor heart rate, provide notifications, and integrate with other health apps.	Offers comprehensive data; integrates with other devices; versatile functions.	Battery life may be limited; can be expensive; may require frequent syncing.	[138]
Wearable sensors	Accelerometers	Devices that measure acceleration forces to track movement patterns, posture, and gait.	Provides detailed movement data; useful for precise monitoring.	Requires careful placement; can be affected by sensor calibration.	[139]
Electromyography (EMG) sensors	Muscle activity monitors	Sensors that measure electrical activity in muscles to assess muscle function and activation patterns during therapy exercises.	Provides insight into muscle activity; aids in optimizing exercise techniques.	It can be uncomfortable to wear; data interpretation may require specialized knowledge.	[140]
Inertial measurement units (IMUs)	Motion tracking devices	Wearable devices combining accelerometers, gyroscopes, and magnetometers to track complex movement patterns and orientation.	Offers comprehensive motion analysis; useful for assessing dynamic movements.	It can be complex to calibrate; data may be affected by device positioning.	[141]
Posture monitors	Posture correction wearables	Devices worn on the back or shoulders to monitor and provide feedback on posture alignment.	Helps improve posture; provides real-time feedback.	May be uncomfortable for extended wear; effectiveness varies by design.	[142]
Smart clothing	Embedded sensors	Clothing is integrated with sensors to monitor physiological parameters like heart rate, breathing rate, and muscle activity during physical activities.	Provides continuous monitoring; unobtrusive and comfortable.	It can be expensive; requires careful maintenance and washing.	[142, 143]
Biometric sensors	Heart rate monitors	Wearable devices that continuously measure heart rate and related cardiovascular metrics during physical activity.	Provides valuable cardiovascular data; useful for monitoring intensity and recovery.	May be affected by device placement; can be uncomfortable for some users.	[144]
Temperature sensors	Thermal monitors	Wearable devices that monitor skin temperature to assess physiological responses to exercise and therapy.	Provides data on body temperature and thermal regulation; useful for evaluating recovery.	Limited to temperature monitoring; it may require additional sensors for comprehensive data collection.	[145]
Feedback devices	Real-time biofeedback	Wearable devices that provide immediate feedback to users about their physiological metrics (e.g., heart rate and muscle tension) to guide exercise performance.	Enhances self-awareness and exercise effectiveness; provides real-time adjustments.	Requires user understanding of feedback; effectiveness depends on device accuracy.	[146]

## Data Availability

The authors have nothing to report.

## Nomenclature

AD: Alzheimer's dementia
AI: artificial intelligence
ASA: automated speech analysis
hCP: hemiplegic cerebral palsy
MAS: Modified Ashworth Scale
MCI: mild cognitive impairment
NLP: natural language processing
PTSD: posttraumatic stress disorder
VR: virtual reality
VR-EBT: VR exposure–based therapy
